# Supplementary material for: Allosteric regulation of enzymatic catalysis by molecular crowding
Source: Commun Chem. 2026 Mar 17;9:172. doi: 10.1038/s42004-026-01977-w (PMC13139598; doi:10.1038/s42004-026-01977-w)
Supplement: Supplementary file 2 — SUPPLEMENTAL MATERIAL [file 42004_2026_1977_MOESM2_ESM.pdf]

Supporting Information for

Allosteric Regulation of Enzymatic Catalysis by Molecular Crowding

Weitong Ren,[†] Jiajun Lu,[‡] Hengyan Huang,[‡] Jian Zhang,[‡] Yuxin Chen,[¶] Zhiqiang Yan,[*,†] Wenfei Li,[*,‡,†,§] and Wei Wang[*,‡]

†*Wenzhou Key Laboratory of Biophysics, Wenzhou Institute, University of Chinese Academy of Sciences, Wenzhou, Zhejiang 325000, China*

‡*School of Physics, National Laboratory of Solid State Microstructure, Nanjing University, Nanjing 210093, China*

¶*Department of Cardiology, Jiangsu Key Laboratory for Cardiovascular Information and Health Engineering Medicine, Nanjing Drum Tower Hospital, Medical School, Nanjing University, Nanjing 210093, P. R. China.*

§*Department of Cardiology, Cardiovascular Disease Center, Jiangsu Key Laboratory for Cardiovascular Information and Health Engineering Medicine, Nanjing Drum Tower Hospital, Medical School, Nanjing University, Nanjing 210093, P. R. China.*

E-mail: zqyan@ucas.ac.cn; wfli@nju.edu.cn; wangwei@nju.edu.cn

Supplementary Methods

Dynamics energy landscape of enzyme catalysis

As mentioned in the main text, We constructed the dynamic energy landscape of AdK by integrating the AICG2+ potential[1,2] and a double-basin energy function[3] using the following formula:

$$
\begin{aligned}
V_{DB}(r, r_1, r_2) =& \frac{1}{2}\left[V_{AICG2+}(r, r_1) + V_{AICG2+}(r, r_2) + \Delta V\right] \\
&- \sqrt{\left(\frac{V_{AICG2+}(r, r_1) - V_{AICG2+}(r, r_2) - \Delta V}{2}\right)^2 + \Delta^2}
\end{aligned}
\tag{1}
$$

$V_{AICG2+}(r, r1)$ and $V_{AICG2+}(r, r2)$ are the single-basin energy function of the apo (open) and holo (closed) form of AdK respectively. r represents the cartesian coordinates of CG beads, r_1 and r_2 represent the reference cooridnates in open and closed states respectively. Δ is a coupling parameter modulating the energy barrier of the transition, controlling the timescale of the protein hopping between two basins. ΔV is associated with the relative energies of the open and closed states, determining the probability of the closed conformation (P_{closed}). In this approach V_{DB} is obtained by analogy with the quantum mechanical treatment of electron transfer. Specifically, a smooth two-state potential is defined as the lower eigenvalue of the characteristic equation:

$$
\begin{pmatrix} V_{AICG2+}(r, r_1) & \Delta \\ \Delta & V_{AICG2+}(r, r_2) + \Delta V \end{pmatrix} \begin{pmatrix} c_1 \\ c_2 \end{pmatrix} = V_{\mathrm{DB}} \begin{pmatrix} c_1 \\ c_2 \end{pmatrix}
\tag{2}
$$

The associated eigenvector (c_1, c_2) also provides a convenient reaction coordinate for monitoring state transitions; we use

$$\begin{aligned}\chi &= ln(\frac{c_2}{c_1}) \\ &= \frac{1}{2}\ln\frac{V_{AICG2+}(r,r_1) - V_{DB}(r)}{V_{AICG2+}(r,r_2) + \Delta V - V_{DB}(r)}\end{aligned} \tag{3}$$

to indicate whether the enzyme resides closer to open or closed state. When $\chi < 0$, the protein is in the open state, whereas $\chi >= 0$ indicates that it is in the closed state roughly. Three double-basin energy terms are employed to describe the interaction of each pair of two domains of AdK. The values of the Δ and ΔV are listed in Table S1.

We categorized the conformations of AdK into 9 states, denoted as XY (where X can be E(empty), T(ATP-bound), or D(ADP-bound), and Y can be E(empty), M(AMP-bound), or D(ADP-bound)), based on the status of the substrates and/or products. This classification was employed to characterize the kinetics of AdK's functional dynamics, encompassing processes such as substrate binding, product release, and the catalytic cycle(see Fig S1). A transition from TM to DD denotes a forward reaction, which is a rapid process in our model. The product release time was defined as the first passage time from the DD state to any state where ADP is not present at any substrate binding sites after the chemical reaction. The substrate binding time was defined as the first passage time to the (ADP-ADP) product state after the product release of the last chemical reaction. Since the time spent in the reaction from TM to DD can be omitted, this definition represents the time of substrate association leading to a successful reaction. Given that substrate binding and product release are closely linked to the enzyme's conformations, this definition of the time for substrate binding and ligand dissociation encompasses the time taken for the large amplitude conformational transition of AdK.

Soft crowder-protein interaction

Although this study primarily focuses on the inert crowding agents, nonspecific interactions between proteins and crowders—such as other proteins or nucleic acids—are additional critical factors influencing enzyme activity. To investigate the impact of these soft crowder agents, we performed additional simulations by introducing an attractive interaction between AdK and crowder particles. To ensure consistency, we employed a piecewise function, as shown in the formula below.

$$V_{pc} = \begin{cases} \varepsilon_{pc}\left(\frac{\sigma_{ref}}{r_{ij}-\sigma_{pc}+\sigma_{ref}}\right)^{12} & r_{ij} \leq \sigma_{pc} \\ \varepsilon_{pc}\left(\frac{\sigma_{ref}}{r_{ij}-\sigma_{pc}+\sigma_{ref}}\right)^{12} + \varepsilon\left[\left(\frac{\sigma_{ref}}{r_{ij}-\sigma_{pc}+\sigma_{ref}}\right)^{12} - \left(\frac{\sigma_{ref}}{r_{ij}-\sigma_{pc}+\sigma_{ref}}\right)^{6}\right] & r_{ij} > \sigma_{pc} \end{cases} \quad (4)$$

The parameter ε_{pc} can adjust the strength of the attractive interaction between protein and crowder, and when ε_{pc} is set to 0, the interaction turns repulsive. σ_{ref} and σ_{pc} are the same as those defined in Eq. 7 of the main text.

Supplementary Table

Table S1: Values of the energy parameter ΔV_i and Δ_i (in kcal/mol) corresponding to various closed conformation populations (P_{closed}) under apo conditions.

P_{closed}	$\Delta V_{\text{LID-CORE}}$	$\Delta V_{\text{NMP-CORE}}$	$\Delta V_{\text{LID-NMP}}$	$\Delta_{\text{LID-CORE}}$	$\Delta_{\text{NMP-CORE}}$	$\Delta_{\text{LID-NMP}}$
0.99	−9.1	−11.5	−5.0	68.0	30.0	10.0
0.73	−6.3	−9.1	−5.0	68.0	30.0	10.0
0.53	−5.7	−8.5	−5.0	68.0	30.0	10.0
0.31	−5.1	−7.9	−5.0	68.0	30.0	10.0
0.15	−4.7	−7.3	−5.0	68.0	30.0	10.0
0.05	−4.2	−6.6	−5.0	68.0	30.0	10.0
0.02	−3.8	−5.9	−5.0	68.0	30.0	10.0
0.007	−3.4	−5.3	−5.0	68.0	30.0	10.0
0.003	−3.1	−4.8	−5.0	68.0	30.0	10.0

Supplementary Figures

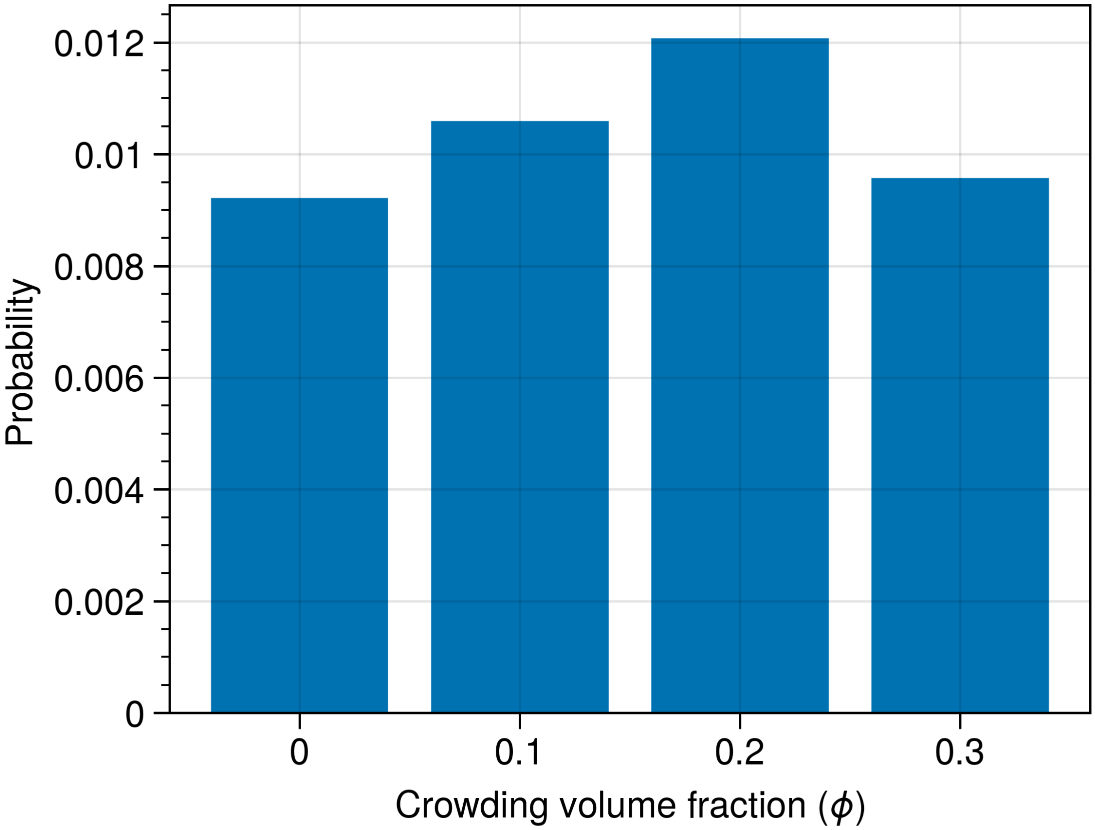

Figure S1: Formation probabilities of the catalytically competent state (substrate-bound closed state) of WT AdK at [ATP] = 9.5 μM under various crowding conditions. The low probabilities primarily reflect the rapid conversion of the catalytically competent state during the reaction cycle.

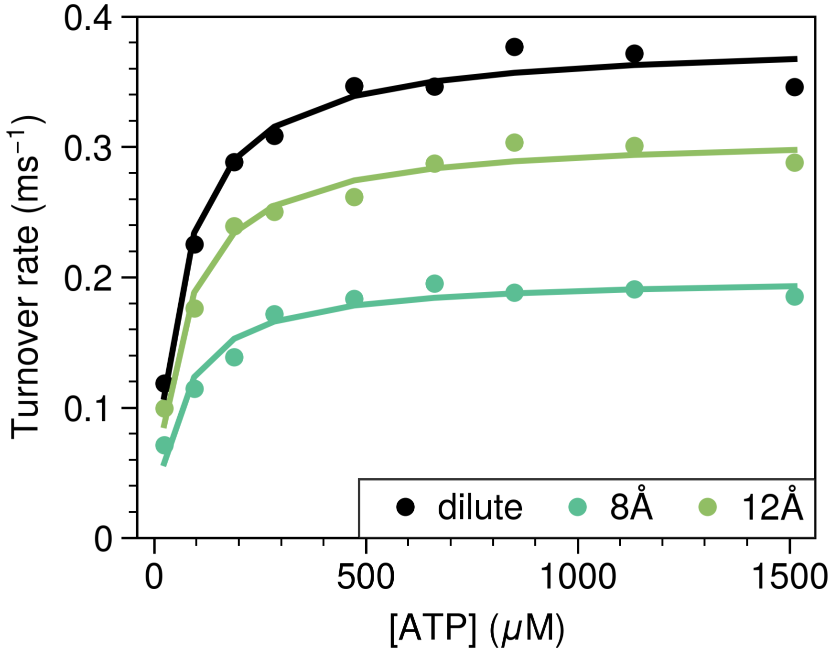

Figure S2: Turnover rate of WT AdK as a function of substrate concentration at $\phi = 0.3$ for different crowder sizes, with the dilute-solution shown for comparison (black line).

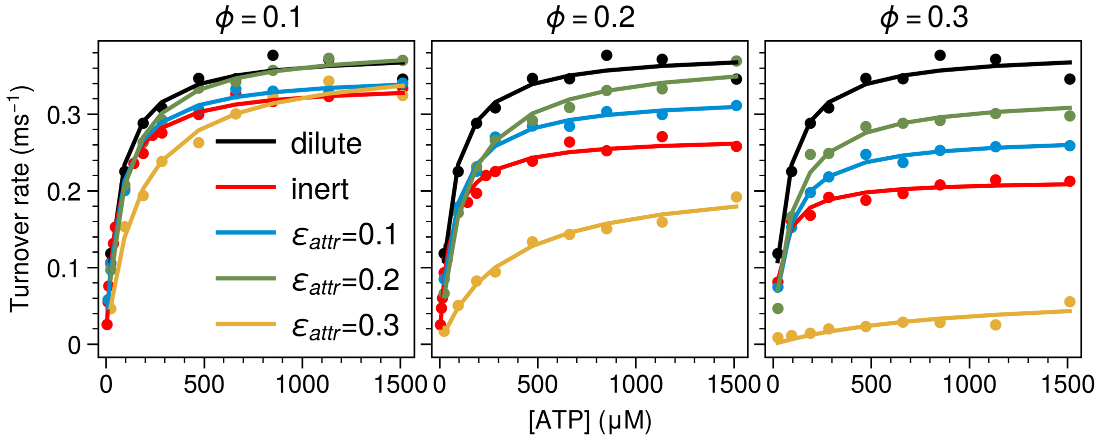

Figure S3: Turnover rate of WT AdK as a function of substrate concentration in soft crowding environments at various volume fractions. Comparison with dilute solution (black line) and inert crowding conditions (red line).

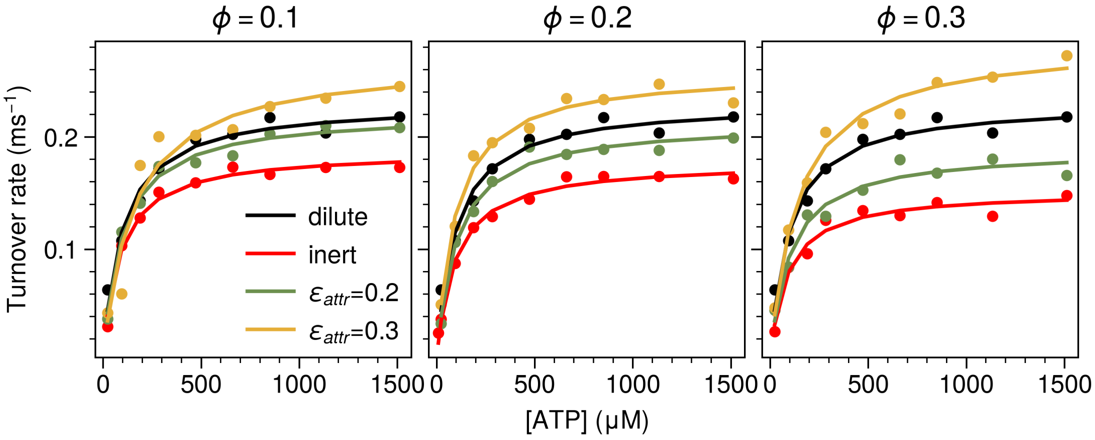

Figure S4: Turnover rate of closed-biased AdK variant($P_{\mathrm{closed}} = 0.99$) as a function of substrate concentration in soft crowding environments at various volume fractions. Comparison with dilute solution (black line) and inert crowding conditions (red line).

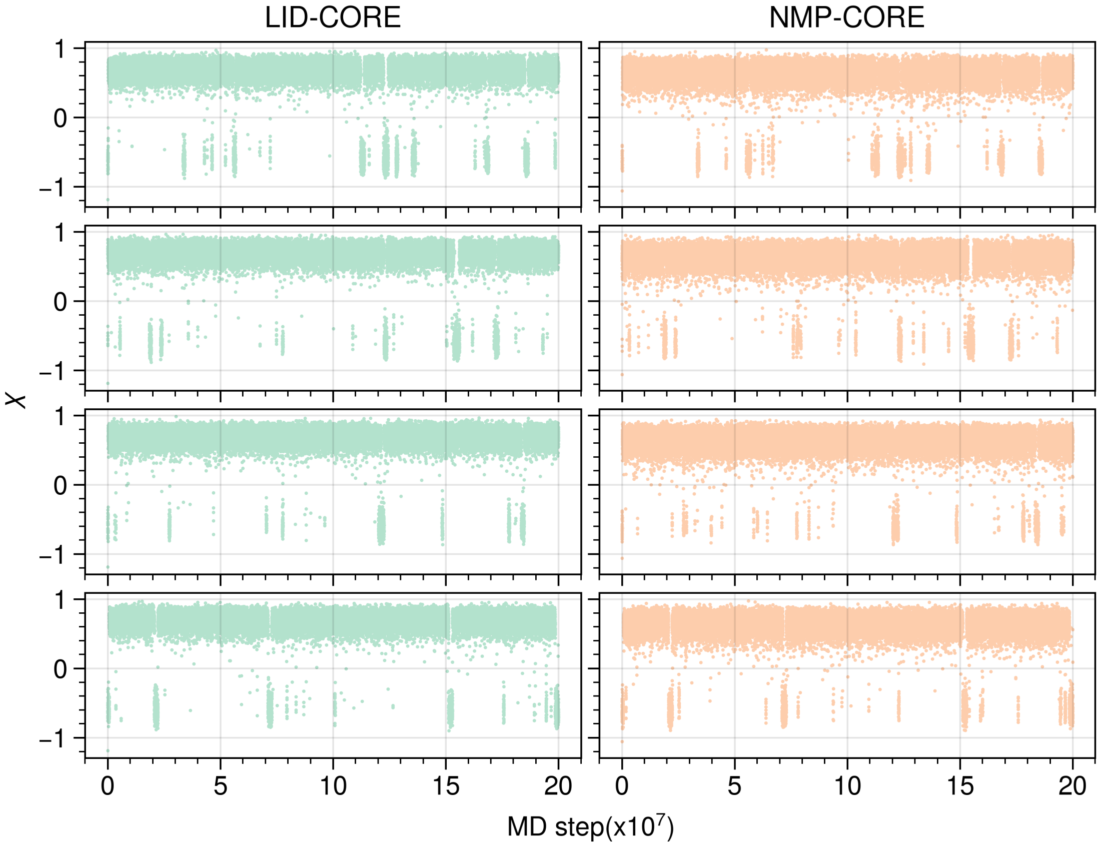

Figure S5: Time evolution of multiple representative trajectories for $\chi_{\mathrm{LID-CORE}}$(left) and $\chi_{\mathrm{NMP-CORE}}$(right) of WT AdK at crowding volume fration of $\phi = 0.3$. $\chi_{\mathrm{LID-CORE}}$ and $\chi_{\mathrm{NMP-CORE}}$ quantify the relative domain motions of LID and NMP with respect to CORE.

References

(1) Li, W.; Wolynes, P. G.; Takada, S. Frustration, specific sequence dependence, and nonlinearity in large-amplitude fluctuations of allosteric proteins. *Proc. Natl. Acad. Sci. U.S.A.* **2011**, *108*, 3504–3509.

(2) Li, W.; Terakawa, T.; Wang, W.; Takada, S. Energy landscape and multiroute folding of topologically complex proteins adenylate kinase and 2ouf-knot. *Proc. Natl. Acad. Sci. U.S.A.* **2012**, *109*, 17789–17794.

(3) Okazaki, K.-i.; Koga, N.; Takada, S.; Onuchic, J. N.; Wolynes, P. G. Multiple-basin energy landscapes for large-amplitude conformational motions of proteins: Structure-based molecular dynamics simulations. *Proc. Natl. Acad. Sci. U.S.A.* **2006**, *103*, 11844–11849.
